# Modelling the impact of school reopening and contact tracing strategies on Covid-19 dynamics in different epidemiologic settings in Brazil

**DOI:** 10.1016/j.gloepi.2022.100094

**Published:** 2022-11-12

**Authors:** Marcelo Eduardo Borges, Leonardo Souto Ferreira, Silas Poloni, Angela Maria Bagattini, Caroline Franco, Michelle Quarti Machado da Rosa, Lorena Mendes Simon, Suzi Alves Camey, Ricardo de Souza Kuchenbecker, Paulo Inácio Prado, José Alexandre Felizola Diniz-Filho, Roberto André Kraenkel, Renato Mendes Coutinho, Cristiana Maria Toscano

**Affiliations:** a Universidade Federal de Goiás, Instituto de Patologia Tropical e Saúde Pública, Rua 235, s/n.°, Setor Leste Universitário, Goiânia, Goiás 74605-050, Brazil; b Instituto de Física Teórica - Universidade Estadual Paulista, Rua Dr. Bento Teobaldo Ferraz, 271, Várzea da Barra Funda, São Paulo, SP 01140-070, Brazil; c Big Data Institute, Li Ka Shing Centre for Health Information and Discovery, Nuffield Department of Medicine, University of Oxford, Old Road Campus, OX3 7LF Oxford, UK; d Departamento de Ecologia, Instituto de Ciências Biológicas, Universidade Federal de Goiás, CP 131, Goiânia, Goiás 74001, Brazil; e Universidade Federal do Rio Grande do Sul, Instituto de Matemática e Estatística, Departamento de Estatística, Avenida Bento Gonçalves, 9500, Agronomia, Porto Alegre, RS 91501-970, Brazil; f Universidade Federal do Rio Grande do Sul, Programa de Pós-graduação em Epidemiologia, Faculdade de Medicina, Campus Saúde, Rua Ramiro Barcelos, 2400, 2° andar, Floresta, Porto Alegre, RS 90035003, Brazil; g Instituto de Biociências - Universidade de São Paulo, A101, Tv. 14, Butantã, São Paulo, SP 05508-090, Brazil; h Centro de Matemática, Computação e Cognição - Universidade Federal do ABC, Avenida dos Estados, 5001, Santa Terezinha, Santo André, SP 09210-580, Brazil

**Keywords:** Decision support techniques, COVID-19, Brazil, Schools, Non-pharmaceutical interventions, Dynamic transmission models

## Abstract

We simulate the impact of school reopening during the COVID-19 pandemic in three major urban centers in Brazil to identify the epidemiological indicators and the best timing for the return of in-school activities and the effect of contact tracing as a mitigation measure. Our goal is to offer guidelines for evidence-based policymaking. We implement an extended SEIR model stratified by age and considering contact networks in different settings – school, home, work, and community, in which the infection transmission rate is affected by various intervention measures. After fitting epidemiological and demographic data, we simulate scenarios with increasing school transmission due to school reopening, and also estimate the number of hospitalization and deaths averted by the implementation of contact tracing. Reopening schools results in a non-linear increase in reported COVID-19 cases and deaths, which is highly dependent on infection and disease incidence at the time of reopening. When contact tracing and quarantining are restricted to school and home settings, a large number of daily tests is required to produce significant effects in reducing the total number of hospitalizations and deaths. Policymakers should carefully consider the epidemiological context and timing regarding the implementation of school closure and return of in-person school activities. While contact tracing strategies prevent new infections within school environments, they alone are not sufficient to avoid significant impacts on community transmission.

## Highlights

• Reopening schools impacts Covid-19 incidence according to context.
• The rate of contact in schools affects the epidemiological dynamics non-linearly.
• Extensive testing is required to reduce cases due to contact tracing significantly.
• Epidemiological context and timing are key to evaluating school reopening.

## Introduction

Among the various non-pharmaceutical interventions (NPIs) recommended to mitigate the COVID-19 pandemic in low and middle-income countries (LMIC), school closures were the most commonly implemented and for the longest period. The additional education and development burden resulting from prolonged school closure is of particular concern, especially when this strategy has been prioritized over other mitigation measures that have been consistently demonstrated and recommended as first-line actions [1]. As such, considering its far-reaching consequences, it has been recommended that school closures should be the last NPI measure to be implemented and the first one to be lifted [2,3].

In particular, the Brazilian population has experienced an unprecedented health crisis due to the COVID-19 pandemic. Previously existing health structure, and health system inequities in the country have been aggravated by the pandemic. In addition, epidemiologic, socio-economic, geographical, and political challenges including uncoordinated actions, variability of public health response among the various states, delayed and insufficient implementation of NPIs, among others [4], coupled with a limited preparedness and emergency response system in place [5], have further impacted the ability to mitigate the pandemic, resulting in one of the highest COVID-19 burdens in the world. With an estimated population of 211 million and being the fifth largest country in territorial extension, Brazil presented, as of April 2021, the second-largest number of COVID-19 deaths worldwide [6].

More than a year after the onset of the pandemic, Brazil ranked first in the world on the duration of school closure [7], having implemented strict school closure policies early on in the pandemic and delayed its reopening [8].

Dynamic transmission modelling has provided evidence to support decision-making related to the timing and impact of various NPI measures, among others [9,10]. It has been recommended that school reopening is followed by large-scale, population-wide testing of symptomatic individuals and effective contact tracing of related contacts, followed by isolation of diagnosed individuals and quarantine of contacts. In Brazil, these large-scale diagnostic testing and contact tracing strategies have remained limited throughout the pandemic [1].

Modelling the dynamics of SARS-CoV-2 transmission in Brazil considering school reopening following the first wave of the epidemic provides an opportunity to evaluate the impact of decisions about school reopening during an epidemic context and produce additional evidence to support policymaking. An inherent challenge in using epidemiological modelling to support decision-making in a country with a geographic scale as large as Brazil is that different locations are subject to decentralized public health policy decisions, besides having distinct demographic profiles and population adherence to NPIs. As different locations can also experience different epidemiological moments, models that support decision-making at the national or state level may not adequately reflect the reality of each place. Thus, more adequate decision-making is benefited from the assessment of the epidemiological realities at local levels. Here, we have modelled the impact of school reopening during the COVID-19 pandemic to identify optimal timing, epidemiological indicators, and the potential benefit of implementing contact tracing in three major urban centers in Brazil that have undergone distinct public health policies, with different demographic profiles, and epidemiological contexts. These results shed light on evidence-based policy implementation to mitigate the impact of the COVID-19 pandemic and offer guidelines for evidence-based policymaking.

Through dynamic transmission modelling, the objectives of this study are: a) to estimate the impact of different levels of schools' reopening in COVID-19 cases and deaths, b) to evaluate the impact of contact tracing strategies during school reopening and whether these could be used to mitigate transmission in the school environment and enable a safer school reopening, c) to identify epidemiological indicators related to the timing of school reopening that are associated with additional disease burden, and d) to contrast the projected impact of school reopening and the true incidence of COVID-19 cases and deaths in 2021 in three major urban centers in Brazil.

## Material and methods

In the following subsections, we first present information on each of the study sites considered in modelling, including demographic and COVID-19 epidemiologic indicators. Next, we provide details of the dynamic transmission model used, the contact tracing and case isolation strategies considered, present model parameters and their sources, and describe the NPIs considered in the model. Finally, we describe the data collection and model calibration processes, explain the school reopening scenarios, and describe sensitivity analyses conducted. For the dynamic transmission modelling, we followed the recommendations of the International Society for Pharmacoeconomics and Outcomes Research (ISPOR) and the Society of Medical Decision Making (SMDM) Modelling Good Research Practices Task Force [11].

### Study sites

Brazil is an upper-middle-income country with >200 million inhabitants, occupying a vast area divided into 5 macro-regions, 27 states, and 5570 municipalities with significant variation of socio-economic, demographic, and geographic patterns. The model considered 3 state capitals, each of them located in different macro-regions of the country - the city of São Paulo-SP in the southeastern region, the city of Porto Alegre-RS in the south region, and the city of Goiânia-GO in the central-west region. São Paulo is the most populous city in South America, has the largest GDP in the country, and is where the first case of COVID-19 was notified in Brazil on February 26th, 2020. Goiânia-GO and Porto Alegre-RS are medium-sized capitals (Table 1).

**Table 1:** Epidemiological and socio-demographic characterization of study sites. Brazil, 2020.

Characteristics	Sites		
	São Paulo, SP	Porto Alegre, RS	Goiânia, GO
Total Population*	12,325,232	1,488,252	1,536,097
Age distribution^&^			
School-aged children^#^			
Childhood Education	689,420	50,835	48,814
Elementary and Middle school	1,387,887	152,868	157,022
High school	388,593	39,371	49,696
Teaching/school professionals^#^			
Childhood Education	53,687	4373	3394
Elementary and Middle school	70,843	8861	8375
High school	29,639	3395	3239
HDI*^£^	0·81	0·81	0·80
GDP per capita (in USD)*	28,940	25,714	16,274
Date of school closure^β^	21-Mar-20	19-Mar-20	18-Mar-20
Incidence of COVID-19 cases at the time of school closure^β^	20·21/100,000 pop.	0·55/100,000 pop.	2·98/100,000 pop.

GDP: Gross Domestic Product; source of variables. HDI: Human Development Index.

*Source: Brazilian Institute of Geography and Statistics (*Instituto Brasileiro de Geografia e Estatística – IBGE*) [12], and converted to US dollars by using the purchase power parity as estimated by the Organization for Economic Co-operation and Development (OECD) (EPPI, 2021) [13] & Source: Population by age in 2020 Source of variables*: Sistema IBGE de Recuperação Automática* – SIDRA (IBGE, 2021) [14].

# *Instituto Nacional de Estudos e Pesquisas Educacionais Anísio Teixeira* (INEP). *Censo escolar*, 2019 (Inep, 2021) [15].

£ Source: national census, 2010 (IBGE, 2021) [14].

β New confirmed cases per 100,000 population per week averaged over a two-week period. Source: Medidas de distanciamento social e evolução da COVID-19 no Brasil (Toscano et al., 2020) [16].

With the onset of the COVID-19 pandemic in 2020, public and private schools were closed in mid-late March, with in-person school activities suspended and shifting to remote online activities, including childhood, elementary, high school, and college education settings. School reopening has been postponed on several occasions, varying over time and by state. The city of Porto Alegre authorized partial school return at the end of September 2020, with little adherence from schools and students. In São Paulo, school reopening was authorized in October 2020. In Goiânia, the reopening was initially authorized for private schools and early childhood education only, and later in November expanded to public schools and all levels of education. In all locations, strict sanitary protocols were in place and a reduced number of students were allowed to be present, requiring rotations among students. The dates schools were closed and reopened, as well as data on COVID-19 cases and deaths over time, by State, are publicly available at the website: https://medidas-COVIDbr-iptsp.shinyapps.io/painel/ [16].

### Epidemiological model

**Fig. 1:**
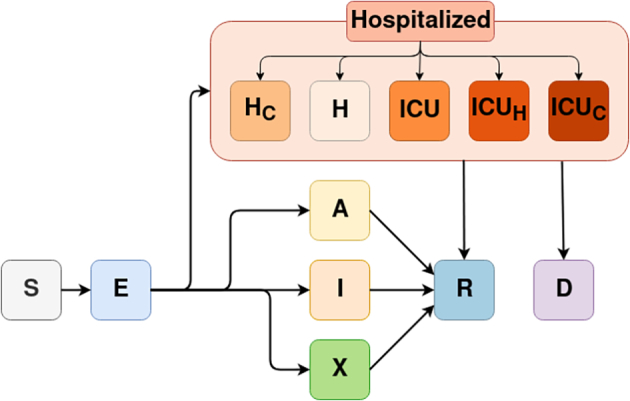
Diagram of the mathematical model and its compartments. Solid arrows describe the possible pathways of individuals in the susceptible (S) compartment after exposure to infection (E), including asymptomatic infection (A), symptomatic infection (I), self-isolation after symptomatic infection (X), and cases requiring hospitalization in regular hospital wards (H) or intensive care unit beds (ICU). If the requirement for hospitalization exceeds the health system capacity, individuals with severe disease move to the compartments of unattended cases requiring hospitalization (H_c_), unattended cases requiring ICU (ICU_h_), or cases requiring ICU treated on hospital wards (ICU_c_). All infected individuals can either recover (R), and hospitalized individuals can either recover (R) or die (D).

**Fig. 2:**
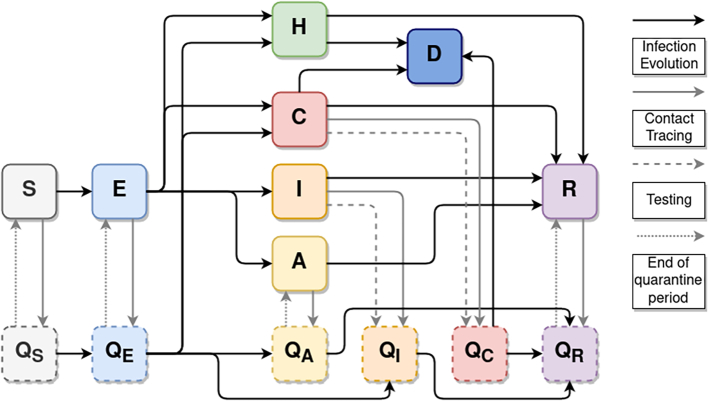
Contact tracing flow diagram. H comprises all hospitalized compartments (*H*, *ICU*, *ICU*_*h*_), and C comprises all critical compartments (*H*_*c*_, *ICU*_*c*_) indicating individuals with severe disease who have not received attendance. Solid black arrows describe the infection pathways in the model, as in Fig. 1. Solid gray arrows describe individuals who are quarantined through contact tracing. Dashed gray arrows describe individuals who are isolated/quarantined because of positive testing in an asymptomatic individual. Dotted gray arrows describe the pathway of individuals after the quarantine period.

We have developed an extended age-structured SEIR compartmental model based on a previously published model [17] that accounts for different levels of disease severity (Fig. 1), also accounting for case isolation and quarantine of contacts (Fig. 1, Fig. 2). The force of SARS-Cov-2 infection transmission is affected by the estimated reduction of contact for each NPI and adherence of NPIs in place. Model compartments are represented in Fig. 1 and a detailed description of the model, including its equations, in the supplementary material, section 2.

From the epidemiological perspective, the progression of infection status and transmission among individuals occurs within different compartments as follows: the susceptible (S) compartment includes individuals without previous exposure to SARS-Cov-2 infection. Once infected, exposed individuals transition to the pre-symptomatic (E) compartment, presenting a relative level of infectiousness. Once fully infectious, individuals may be still asymptomatic (A) or become symptomatic (I). A fraction of symptomatic individuals may self-isolate themselves (X) to decrease the risk of infecting others. Individuals from the asymptomatic (A), symptomatic (I), and self-isolated (X) compartments will transition to the Recovered (R) compartment after the infectious period. Alternatively, infected individuals with more severe diseases (ie., requiring hospitalization) transition to the hospitalization compartments, which include either hospitalization in regular hospital wards (H) or intensive care units (ICU). If the demand for hospital and ICU beds exceeds the number of available beds, as in the case of a health system collapse, unattended individuals transition to the compartments H_c_ (unattended cases requiring hospitalization) and ICU_c_ (unattended cases requiring ICU), respectively. Furthermore, those requiring ICU beds can be managed in hospital beds (ICU_h_) if that is the only option available. Individuals in those compartments will transition to the Recovered (R) or Deceased (D) compartments. We assume that all deaths due to COVID-19 will occur in hospitalized individuals and that no deaths occur in symptomatic individuals not requiring hospitalization.

We divided the population strata into 5 year age sub-groups, ranging from 0 to 95 years and over. Contacts among individuals can occur in four different settings: work, home, school, and in the community (i.e. public transportation, social gatherings, shopping activities, etc.). The contact rate between individuals by age group and by settings type are based on the contact matrices for the Brazilian population in urban environments (see supplementary material, section 2). The infection transmission probability is derived from the pattern of social contacts and the frequency of potential contacts, varying by age group and the setting where the contact occurs.

In addition, the infection transmission rate can be affected by the following NPIs: self-isolation, social distancing, use of masks, home-office, cocooning of older adults, and school closure (see supplementary material, section 3).

### Model parameters, data source, and fitting

Model parameters considered national information systems and the best available evidence from the literature (Table 2, see also the supplementary material, section 4). Upon the lack of data from the literature, some of the parameters were assumed based on reasonable values.

**Table 2:** Model parameters considered in the analysis of COVID-19 school reopening in Brazil, 2020.

Symbol	Description	Value	Source
*rho*	Relative infectiousness of incubation phase	10·5%	[18]
*rhos*	Relative percentage of regular daily contacts when hospitalized	10%	Assumed
*omega*	Average duration of immunity	∞	Assumed
*gamma*^*−1*^	Average of incubation period	5·8 days	[19]
*nui*^*−1*^	Average duration of the symptomatic infection period	9 days	[20]
*pclin*	Probability upon infection of developing clinical symptoms by age groups		
	0–19 years	30·5%	[21]
	20–59 years	56%	[22]
	60 or more years	69%	[22]
*scale ihr*	Scaling factor for infection hospitalization rate	0·8	Fitting
*mask eff*	Estimated reduction of contact due to mask use	85%	[23]
*selfis eff*	Estimated reduction of contact due to self-isolation if symptomatic	80%	Assumed
*mask cov*	Adherence to mask usage	Varies by study site and over time	See SM sections 3, 4, and 5
*selfis cov*	Adherence to self-isolation	Varies by study site and over time	See SM sections 3, 4, and 5
*dist cov*	Adherence to social distancing at the community level	Varies study site and over time	See SM sections 3, 4, and 5
*work cov*	Adherence to home-office	Varies study site and over time	See SM sections 3, 4, and 5
*school cov*	Adherence to online (not in-person) school activities	Varies study site and over time	See SM sections 3, 4, and 5
*cocoon cov*	Adherence to cocooning of older adults	Varies study site and over time	See SM sections 3, 4, and 5
*dist eff*	Reduction of contacts in the community among those adhering to social distancing	95%	Assumed
*work eff*	Reduction of contacts at work among those adhering to home-office	95%	Assumed
*school eff*	Reduction of contacts in school upon school closure	100%	Assumed
*cocoon eff*	Reduction of contacts with older adults in all settings as a result of cocooning older adults	90%	Assumed
*report*	Percentage of all asymptomatic infections that are reported	0%	Assumed
*reporth*	Percentage of non-severe hospitalizations that are appropriately treated	95	Assumed
*reportc*	Percentage of all symptomatic infections that are reported	1%	Assumed
*give*	System capacity stressor	65%	Assumed

SM: Supplementary Material.

The effectiveness of each NPIs considered in the model is dependent on the population adherence (*NPI*_*cov (t)*_ **∈** {0,1}) and estimated reduction of contacts (*NPI*_*eff*_ **∈** {0,1}) for each intervention, each varying from 0 to 1 (0–100%). Adherence to each intervention by study site was based on the adherence of the population to each NPI in 2020 and was obtained from monitoring NPI implementation and level of strictness over time [4] (see supplementary material, sections 3–5). The reduction of contacts (in %) refers to the percentage of potential contacts in a given location that is prevented as a result of the intervention and was assumed as fixed over time (Table 2).

For each study site, length of stay in hospital wards and ICU, and Intra-Hospital Fatality Rate (IHFR) were obtained from Brazil's Epidemiological Surveillance Information System for Acute Respiratory Illness (SIVEP) (see further details on the supplementary material, section 4).

Fitting was based on the number of COVID-19 hospitalizations and deaths reported to the SIVEP database (see additional details on the supplementary material, section 4).

### Time horizon and analytic framework

School reopening intervention in the model was set at February 1st, 2021, the scheduled date for reopening school in most Brazilian states, following summer holidays in December 2020 and January 2021. Incidence of COVID-19 cases and deaths was projected throughout 2021, until December.

### School reopening scenarios

We evaluated the effect of reopening schools by simulating scenarios considering increasing values of the percentage of potential contacts of individuals in the school setting (henceforth described as PCS) after reopening schools. The scenarios consider the parameter “adherence to online (not in-person) school activities”, and it is defined as 100 minus the *school cov* parameter (i.e. 100 – *school cov* parameter), in percentage. This approach can reflect a combination of strategies that reduce the likelihood of contact and transmission in a school environment, including but not limited to the implementation of hybrid learning approaches (online and in-person), and infection prevention measures when returning to school such as limiting the maximum number of students in the classroom, natural ventilation of indoor spaces, a distance of at least 6 ft between students and teachers/staff, mask use at school, among others. We implemented these scenarios by ranging values from 0 to 100% at regular intervals of 20, and considering that they started on February 1st, 2021.

To assess solely the effect of school reopening, we assumed that other interventions remained unchanged and compared the weekly incidence of new COVID-19 cases and deaths throughout the year 2021. We also evaluated the additional incidence of cases and deaths due exclusively to school reopening in three age groups: young (<20 years old) adults (between 20 and 59 years old), and older adults (>60 years old). This value was estimated by the difference in the cumulative incidence of cases and deaths on December 31, 2021, compared to the baseline scenario where schools remain closed for the whole year (PCS = 0).

### Diagnostic testing, contact tracing, isolation, and quarantine scenarios

We also evaluated the effects of case isolation, contact tracing, and quarantining contacts in schools upon reopening (henceforth described as CT model), to mitigate transmission events within the school environment. This consists of identifying symptomatic individuals and their contacts, isolating positive cases, and quarantining contacts who tested positive for COVID-19 after diagnostic testing. Since in Brazil diagnostic testing has been prioritized for symptomatic hospitalized individuals, our contact tracing model prioritizes the usage of the available tests for severe and symptomatic individuals and, if still available, it proceeds to test asymptomatic individuals. The CT model is further described in the supplementary material, section 2.

Fig. 2 presents the flow diagram of the model compartments and quarantined compartments considered. Symptomatic or asymptomatic (not hospitalized) individuals who test positive are isolated at home, and are transferred to the corresponding quarantine compartment (Q, with the respective sub-index Individuals in non-infected compartments (S and R) who have contact (modelled through contact rates among compartments and age groups) with individuals who tested positive are quarantined, and are also transferred to the corresponding Q compartment. In both situations, only a fraction of individuals (the adherence to the intervention) follows the isolation or quarantine procedure.) In these compartments, contacts are restricted to their households. Symptomatic individuals are isolated until recovery, here depicted as quarantine of infected individuals. Asymptomatic infected individuals are kept in quarantine until the end of the isolation/quarantine period (ten days). Note that regardless of the contact tracing strategy, symptomatic individuals may already self-isolate themselves according to the adherence to the self-isolation intervention.

As our main objective was to assess whether contact tracing strategies can contribute to mitigating infection transmission in the school environment, and potentially increase the safety of reopening schools, we only considered CT strategies restricted to the household and school environments.

Finally, for each scenario, we estimated the reduction in the final cumulative incidence of COVID-19 cases and deaths after the implementation of contact tracing and isolation strategies for the period of simulation. For each scenario of PCS, we simulated daily tests for the population proportional to each city's population size, until increasing the number of tests no longer reduced the incidence of cases and deaths. The use of the contact tracing strategy starts on the same date as the school reopening starts in our model. We assumed that all individuals that became contacts are timely traced and isolated, thus, the effectiveness of the strategies depends on the number of daily tests available for use in the population.

### Sensitivity analysis

To account for both parameter and model uncertainties, we performed a sensitivity analysis varying values for the following nine parameters: relative infectiousness of incubation phase (*rho*), relative percentage of regular daily contacts when hospitalized (*rhos*), probability upon infection of developing clinical symptoms for young individuals (*pclin young*), estimated reduction of contact due to mask use (*mask eff*), estimated reduction of contact due to self-isolation if symptomatic (*self eff*), adherence to self-isolation (*selfis cov*), adherence to social distancing at the community level (*dist cov*), adherence to home-office (*work cov*), and adherence to cocooning of older adults (*cocoon cov*). Since changes in the value of a given parameter evaluated by the sensitivity analysis require a new estimation for the free parameters of the model, we performed a new fitting including the parameter evaluated in the sensitivity analysis (see details on the supplementary material, section 5), for the same period used for the previous fitting in each city. We then used the new fitted values to simulate the school reopening scenarios and compared the additional incidence of cases and deaths due to school reopening relative to the model with the original values of the parameter chosen for the sensitivity analysis.

### Contrasting modelled and true incidence of events after school reopening

As we modelled only school reopening with additional combined contact tracing strategies, without accounting for other epidemiologic patterns of disease progression which at that time were not amenable to modelling, particularly the emergence and circulation of variants of concern with different transmissibility patterns, and unanticipated relaxation of other NPIs by policymakers, we also contrasted the projected COVID-19 incident cases and deaths over time after school reopening, and the true epidemic curves of COVID-19 cases, hospitalization, and deaths in the study sites, considering the same analytical horizon.

## Results

### School reopening

**Fig. 3:**
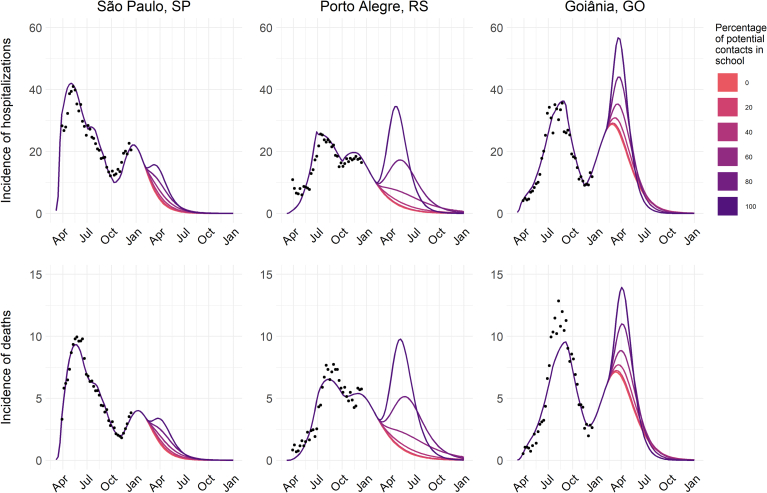
Epidemiological dynamics for different scenarios of increase in the percentage of potential contacts in school for three Brazilian capitals: São Paulo, Porto Alegre, and Goiânia. Colors represent the percentage of potential contacts of individuals in the school setting after reopening schools on February 1st, and dots represent the hospitalization and death data from the SIVEP database.

Our model showed that school reopening results in an increase in the number of new COVID-19 cases and related deaths proportional to the degree of increase of potential contacts in the school environment (Fig. 3). The estimated increase is affected by local epidemiological indicators, particularly the incidence and trend of COVID-19 cases and deaths, at the moment of reopening in each study site. As shown in Fig. 3, in settings of high disease occurrence, the impact of school reopening is projected to be more significant.

In a scenario where schools remain closed throughout 2021 and other interventions unchanged, both São Paulo and Porto Alegre were expected to present a decline in the number of cases with the epidemic under control after the second semester of that year. However, the rate of decrease in the number of cases is reduced in scenarios with a gradual increase in transmissibility in schools (for instance, PCS ⩽ 60% in Porto Alegre, and PCS ⩽ 80% in São Paulo). In scenarios where PCS exceeds these values, the effects of increasing transmission within schools can reverse the trend and lead to a third surge in the number of cases in both capitals. Goiânia, on the other hand, by the end of 2020 was experiencing a second surge in the number of cases. An increase in transmission in the school environment is predicted to sustain the growth of cases for a longer time proportional to PCS: while small PCS values (⩽ 40%) result in small increases in the number of cases, higher PCS values (> 60%) may lead to a daily number of cases and deaths close or even higher than observed in the first peak of cases.

**Fig. 4:**
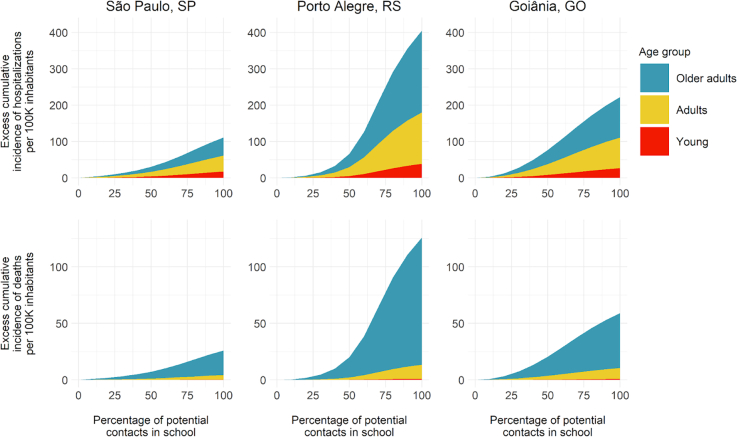
Excess cumulative incidence of COVID-19 hospitalizations and deaths per 100 k inhabitants according to the percentage of potential contacts in schools after school reopening.

School transmission also resulted in a non-linear increase in the excess cumulative incidence of COVID-19 cases and deaths in all study sites, affecting age groups disproportionately (Fig. 4). The excess cumulative incidence represents the additional incident cases and deaths projected by the end of 2021, compared to the baseline scenario where schools remain closed throughout the year, computed for each age group.

São Paulo had the smallest excess incidence in cases and deaths with increasing PCS, reaching approximately 100 cases per 100 k inhabitants and 25 deaths per 100 k inhabitants. On the other hand, Porto Alegre had a considerable increase in incidence, reaching 400 cases per 100 inhabitants and 120 deaths per 100 k inhabitants. With PCS values above 50%, Porto Alegre shows a substantial increase in the incidence of cases and deaths. Goiânia presented an intermediate value among the other capitals, reaching just over 200 cases per 100 k inhabitants with the total reopening of schools, and a little over 50 deaths per 100 k inhabitants. In general, while PCS equal to or below 30% had only a marginal effect on the outcomes, levels of school reopening above this limit resulted in greater increases in the final number of cases and deaths. Despite most contacts on the transmission in school occurring between individuals of young age, by the end of the simulations, they represent <12% of cases in all capitals and <1% of deaths. On the other hand, older adults comprise a substantial portion of the population infected due to school reopening, representing nearly half of the cases and >80% of deaths by the end of the period.

### Modelling case isolation, contact tracing, and quarantining scenarios

**Fig. 5:**
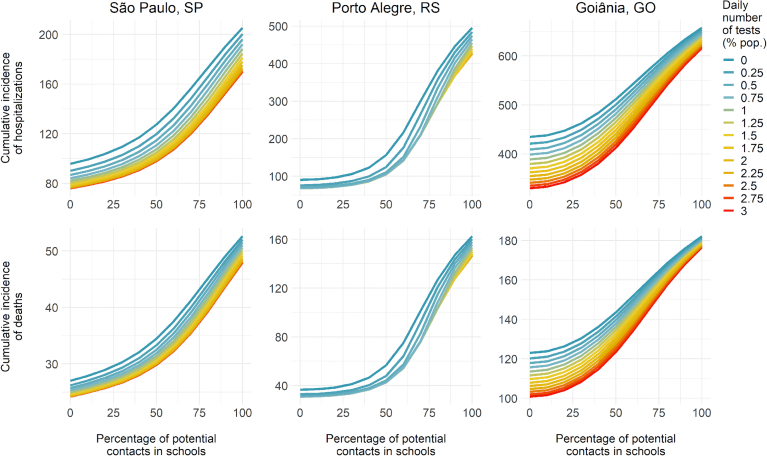
Effects of implementing case isolation, contact tracing, and quarantining contacts in schools on the cumulative incidence of COVID-19 cases and deaths, by the daily number of tests available and scenarios of school reopening (percentage of potential contacts in schools). The projected cumulative incidence of cases and deaths presented is for the period from school reopening (February 1st, 2021) to the end of 2021 (December 31, 2021).

Finally, our simulations indicate that the implementation of case isolation, contact tracing, and quarantining contacts can reduce the incidence of COVID-19 cases and deaths throughout the pandemic, and particularly upon school reopening. Nonetheless, a significant number of daily tests are required to significantly impact the incidence of COVID-19 cases and deaths (Fig. 5). After a certain threshold, increased testing capacity is no longer able to reduce the incidence of cases or deaths. In São Paulo and Goiânia, a daily number of tests corresponding to 3% of the population is required to result in the maximum reduction of cases and deaths due to the isolation of infected individuals. However, while in São Paulo the decrease in the incidence of cases and deaths is independent of PCS, in Goiânia the effectiveness of this measure is greater for lower levels of school reopening (i.e. lower PCS values) but decreases when PCS reaches its maximum value. In Porto Alegre, a testing rate representing 0·75% of its population is required to achieve the maximum reduction in the number of cases if PCS is below 75%, and up to 2% above that value.

### Sensitivity analysis

**Fig. 6:**
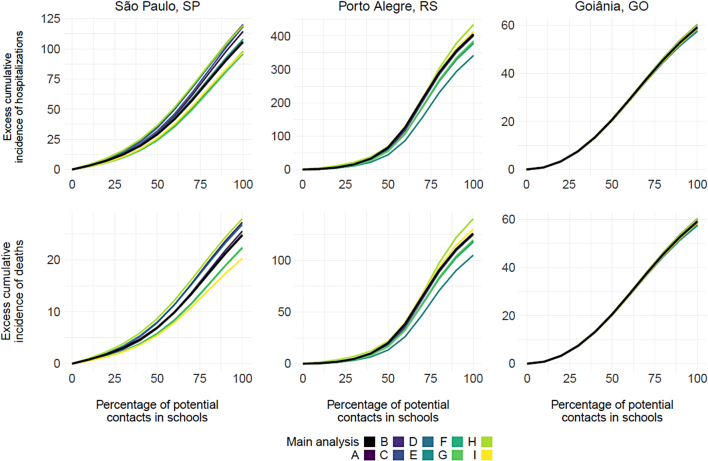
Results of Sensitivity Analysis presenting excess incidence of hospitalizations and deaths according to the percentage of potential contacts in schools after school reopening, when compared to fully closed schools. Different lines represent the models simulated with the parameters fitted on the sensitivity analysis. A; Relative infectiousness of incubation phase (*rho*); B. Relative percentage of regular daily contacts when hospitalized (*rhos*); C. Probability upon infection of developing clinical symptoms for young individuals (*pclin young*); D. Estimated reduction of contact due to mask use (*mask eff*); E. Estimated reduction of contact due to self-isolation if symptomatic (*self eff*); F. Adherence to self-isolation (*selfis cov*); G. Adherence to social distancing at the community level (*dist cov*); H. Adherence to home-office (*work cov*); and I. Adherence to cocooning of older adults (*cocoon cov*).

For the sensitivity analysis, we evaluated how changes in a parameter of interest can qualitatively and quantitatively alter the simulation results for the different scenarios evaluated for the reopening of schools. Thus, we compared the final difference in the incidence of cases and deaths to a baseline scenario without school reopening. The simulations were repeated for the different school reopening values (PCS) evaluated in the school reopening scenario and compared with the original simulation (supplementary material, section 5). As shown in Fig. 6, there is a small variation between the difference in the incidence of cases and deaths for the main results and the outcomes of the model for the sensitivity analysis for the different parameters. In Goiânia, the incidence of cases and deaths is close to the original projections for all PCS values. In São Paulo and Porto Alegre, increasing PCS results in greater variation in the final incidence projections, but the qualitative aspects remain similar to the main results.

### Modelled incidence observed incidence

**Fig. 7:**
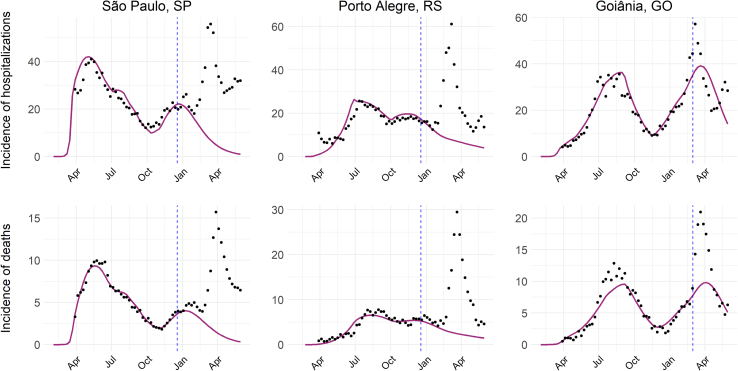
Epidemic curves of hospitalization and deaths for São Paulo, Porto Alegre, and Goiânia. Dots represent the incidence reported for each city. Purple line: simulation from the model for the worst school reopening scenario, in which schools are fully reopened after March 1st, 2021. Dashed blue line: maximum date used for fitting. (For interpretation of the references to colour in this figure legend, the reader is referred to the web version of this article.)

When contrasting the projected COVID-19 incident cases and deaths over time after school reopening, and the actual epidemic curves of COVID-19 cases, hospitalization, and deaths in the study sites, the latter strongly surpassed the original projections (Fig. 7). As of April 2021, in all study sites, the third wave of the pandemic was observed. It was mostly driven by the emergence and predominance of the Gamma variant, which was first reported in the country in December 2020 in the Northern Region (Manaus), and then disseminated to the whole country. This variant is more transmissible and partially escapes from prior infection and vaccine-induced immunity. Added to this unforeseeable event, several states progressively lifted NPIs early in 2021, considering past trends of disease and confidence in future vaccination.

## Discussion

Among the various recommended non-pharmaceutical interventions to reduce transmission and mitigate COVID-19 pandemic, school closure has been adopted globally, mainly during the first wave of the pandemic in early 2020. In addition to the loss of learning, higher exposure to domestic violence and child abuse, lack of access to meals and immunization delivered at school, social and emotional impacts, among others, are potential impacts of school closures [24].

Although most developed countries gradually reopened schools, implementing strict in-school transmission prevention measures, this did not happen in developing and low-income countries in the first and most of the second year after the beginning of the pandemic. In these countries, schools remained closed for a very long time, with severe short and long-term implications. These are also countries where access to vaccines is limited and vaccination rollout is slower, posing additional challenges to safe school reopening.

School closures were among the first actions taken early in the pandemic in most Brazilian states, even before demonstrated community transmission of SARS-CoV-2 [4]. Further, Brazil delayed the reopening of schools and stands among the countries in which schools remained closed for the most prolonged period since its inception in 2020.

Although the school closure was one of the few interventions adopted at national level, the implementation of this measure did not occur without strong controversies, with different social actors advocating for the return of school activities. At the same time, especially in the first two years of the COVID-19 pandemic in Brazil, there was little evidence or studies that addressed the epidemiological consequences of this reopening within the Brazilian reality. In particular, until early 2021, the only studies evaluating the effect of reopening schools were restricted to the UK [19,20]. However, extrapolating the results to this country may not be adequate, considering the difference how NPIs were adopted in each country, the differences in geographic scales, as well as the demographic profile and access to health services by the population of these countries. Especially in Brazil, the difficulty of comparison is added to the fact that even between different cities these characteristics can vary considerably so that the adherence or relaxing of the same NPI can result in different effects according to the reality of each location.

In this study, we sought to overcome some of these difficulties by considering in our model the joint effect of different NPIs implemented/in place, demographic characterization, and epidemiological situation in each city. The latter considered the incidence of COVID-19 hospitalizations and deaths, and infection transmission rates were proportional to contact rates between different age groups in different settings. Furthermore, our model incorporates the potential effect of the collapse of the network of contacts in a residential setting [10], which allows a better fitting of the parameters used in our model.

Considering the continental dimensions and regional specificities of Brazil, it was expected that the epidemiologic pattern of COVID-19 progression would vary significantly among the country's 27 states, as it did in 2020. This was further challenged by a struggle to define responsibilities at all levels of government (municipal, state, and federal), coupled with erratic and many times misguided communication strategies [25]. In the absence of coordinated and equitable responses, the epidemic in Brazil resulted in high and unequal infection and mortality burdens [26].

During the first wave of infection in Brazil, COVID-19 cases and deaths were mainly concentrated in large metropolitan areas, but nonetheless, schools in the whole country were closed and remained closed throughout the year 2020. This provided an opportunity to model school reopening in different metropolitan areas with varying epidemiologic, socio-economic, and demographic characteristics, mimicking different LMIC settings facing similar challenges. Our results provide valuable evidence on the potential impact of school reopening during the pandemic and provide insights on when and how to reopen schools more safely in LMIC settings.

First, we demonstrate that the impact of school reopening in terms of incident COVID-19 cases and deaths is small if the potential contacts in school upon reopening are lower than 40%, becoming more significant when contacts are increased by 60% and more. This reinforces the various epidemiologic evidence and recommendations of the need to implement strict prevention and social distancing measures within schools, and not allow for in-person activities for all students at once.

Second, the magnitude of the impact of school reopening in new cases and deaths is highly dependent on its timing. In settings with declining trends in COVID-19 incidence, small impacts were projected after school reopening (as observed in São Paulo), whereas, in settings with increasing trends, the magnitude of the projected impact was more significant (as projected in Goiania). Modelling simulations that evaluate the timing of school closure to suppress the epidemic also demonstrate that the timing of implementation of this intervention result in distinct outcomes on the epidemic curve [27]. In addition, the set of other interventions and the level of community transmission in other settings can have significant impacts on the result of school reopenings, as has also been demonstrated in other studies [[28], [29], [30]].

Third, the excess incidence of COVID-19 cases and deaths is most significant in the older adults, a high-risk group for COVID-19 complications and death. Although school reopening leads initially to a higher level of transmission in younger individuals, the mixing between these groups will eventually lead to infection in older adults, which present higher chances of hospitalization and death. Our results agree with other projections that indicate the school reopening leads to more deaths when compared to a scenario without school reopening [28,31]. This finding suggests that upon school reopening, vulnerable populations such as older adults must be protected and isolated to minimize the potential impact.

Finally, large-scale testing of suspected individuals and contact tracing strategies are important to minimize the impact of school reopening, and more effective when combined with other mitigation strategies, similar to the predictions found for other countries [[32], [33], [34], [35]]. However, our simulations indicate that they require a significant number of tests to do so, especially if there are already high levels of community transmission. Unfortunately, most countries where schools have been closed the longest are also countries that struggle to access diagnostic tests, have limited personnel in place to adequately implement contact tracing strategies, and are thus less likely to have these strategies implemented.

Varying parameters did not impact our results, as demonstrated in sensitivity analysis, pointing to the robustness of our model and parameter estimates.

In summary, we demonstrate that the impact of reopening schools varies in different settings, primarily due to the timing regarding the epidemic curve at the time of reopening but also due to the percentage of potential contacts in school. We considered data adjusted for the period from March through December 2020, and modelled school reopening occurring hypothetically in February 2021. As observed in Goiania, where the epidemic curve was rising at this time, even with a small percentage of potential contacts at school we observed a new surge. Alternatively, in São Paulo and Porto Alegre, where the curve was declining, it could be possible to reopen schools with a high percentage of potential contacts, 80% and 60% to São Paulo and Porto Alegre, respectively. Therefore, considering the epidemiological scenario is essential to decide when reopening schools and what percentage of potential contacts are allowed.

It is noteworthy that our modelling was conducted before the circulation of the gamma variant in Brazil. Despite the reports of gamma variant circulating in a restricted area of the country at that time (Manaus, in the Amazon Region), it was not possible to model a scenario of school reopening in the context of gamma variant predominance. Since early 2021, the gamma variant emerged and disseminated throughout the country, quickly becoming the predominant circulating variant in all states [36]. This culminated in synchronic waves of disease in most states of the country in April 2021, and an overload of the already-stretched healthcare system, resulting in a current collapse of the country's health services and increased mortality from COVID-19 and other causes [37].

School reopening was further delayed as a result and only by the end of May 2021, schools indeed reopened in most states in the country. Nonetheless, when contrasting the projected impact of school reopening with the additional COVID-19 burden as a result of the third wave of disease associated with the gamma variant emergence and dissemination, in the first quarter of 2021, the estimated impact of school reopening was negligible (Fig. 7).

Interestingly, when schools did reopen by the end of May 2021, differently than what had been predicted by several studies [38,39], we did not observe a substantial increase in cases and COVID-19 related mortality. We hypothesize that this might result from the fact that school reopening might have represented a marginal impact on new COVID-19 cases and deaths in an epidemic context in which the other measures of social distancing had already been relaxed or lifted.

## Conclusions

Our results provide valuable evidence to policymakers, particularly regarding the safe opening of schools. We recommend that a safer school reopening in an epidemic context should take into consideration the social distancing measures in place, occur at an adequate timing (i.e., when infection transmission in the community is low), progressively (i.e., not bringing all children to in-person activities at once) and associated with strong diagnostic and contact tracing programs in the communities. School reopening must be adapted to the local epidemiologic context and be guided by epidemiologic indicators and should be prioritized and among lifting different non-pharmaceutical interventions during the COVID-19 pandemic.

## Declaration of Competing Interest

The authors declare that they have no known competing financial interests or personal relationships that could have appeared to influence the work reported in this paper.

## Appendix A Supplementary data

Supplementary material Image 4
